# Correction: Spatial pattern and associated factors of timely vaccination in Ethiopia using EDHS-2016 data: A multilevel and spatial analysis

**DOI:** 10.1371/journal.pone.0300482

**Published:** 2024-03-07

**Authors:** Muluken Chanie Agimas, Meron Asmamaw Alemayehu, Nebiyu Mekonnen Derseh, Fantu Mamo Aragaw, Daniel Alayu Shewaye

The second, third, and fourth authors’ names are spelled incorrectly. The correct names are: Meron Asmamaw Alemayehu, Nebiyu Mekonnen Derseh, and Fantu Mamo Aragaw, respectively. The correct citation is: Agimas MC, Alemayehu MA, Derseh NM, Aragaw FM, Shewaye DA (2024) Spatial pattern and associated factors of timely vaccination in Ethiopia using EDHS-2016 data: A multilevel and spatial analysis. PLoS ONE 19(2): e0296123. https://doi.org/10.1371/journal.pone.0296123.
