# Supplementary material for: Spatial pattern and associated factors of timely vaccination in Ethiopia using EDHS-2016 data: A multilevel and spatial analysis
Source: PLoS One. 2024 Feb 7;19(2):e0296123. doi: 10.1371/journal.pone.0296123 (PMC10849225; doi:10.1371/journal.pone.0296123)
Supplement: Attachment — Submitted filename: Comments to authors-1.docx [file pone.0296123.s001.docx]

Comments to author(s)

Manuscript Number: PONE-D-23-00874

Title: Spatial variation and determinants of age-appropriate vaccination practice in Ethiopia using EDHS 2016 data: A multilevel and spatial analysis

I appreciate the authors for their effort to conduct this study. The manuscript reports findings based on the objective of the study. The overall organization of the manuscript is also good. But, there are some concerns that need corrections. The comments are listed below, review and amend them.

Introduction

1. Some of your references cited in the introduction part has no relation to your document.
   a. Citation #1 talks about “The impact of the vaccination program for hemorrhagic fever with renal syndrome in Hu County, China”, remove this citation and replace it with appropriate citation.
   b. Citation #2 briefs about influenza vaccine not about period of vaccine administration. Revise and replace with the appropriate citations.
   c. Citation #3 is not the finding of the research you cited. Rather, you used from cross reference. So, replace it with appropriate source,
2. Page 3, line 58-59; revise the sentence. Next to citation 6, please add the program of vaccination based on WHO recommendation.
3. Page 4, line 71; write full words on their first appearance, VPDs.
4. Page 4, line 76; the citation used for Toke kutaye reference number 18 is not correct. The reference listed on number 18 is Xiao X, Wu Z-C, Chou K-C , 2011.

Methods

5. Page 4, line 84-85; the population of Ethiopia is 114,963,588, include your reference
6. Page 4, line 89-90; what is the need of this statement “the Ethiopian demographic health survey of 2016 revealed that among ten children aged 12-23, four children (39%) received all the basic vaccines (19).:”?
7. Page 6, line 119; table 1, there is no table clearly showing table 1 since table cations are 1 for all.

Results

8. Page 7, line 144, line 149, and page 9 line 200; you cited table 2,3, and 4, but not appropriately used the captions.
9. Page 8, line 162; figure 4 is not available in lists of tables or has no caption.
10. Pages 7- 8, line 158-162; why you included about anemia? Remove it.

Discussion

11. At the end of your discussion add strength and limitation of your study.

Conclusion

12. Actually, you have no variable that shows lack of transportation fees. So, you could not give the following recommendation since it is not a part of your findings. So remove it. “provide outreach vaccination for those are difficulty accessing the vaccine because of lack of transport fees could bring a significant contribution to improving age appropriate vaccination practice by health care workers.”
13. Page 12, line 264-266; move the strength and limitation to discussion part.

References

14. References 1-3 are not related with your citation, so change and update them.
15. Reference number 19 is not fully written. Revise it.

Tables

16. There are 4 tables listed in the manuscript. But the caption of each table is not correct. All tables are labeled as “table 1”, revise and correct them.
17. Sociodemographic table, under variable region, change Somalia to Somali.

Figures

18. Out of the 7 figures listed in in this manuscript, one figure has no caption. Revise it.
