# Supplementary material for: Spatial pattern and associated factors of timely vaccination in Ethiopia using EDHS-2016 data: A multilevel and spatial analysis
Source: PLoS One. 2024 Feb 7;19(2):e0296123. doi: 10.1371/journal.pone.0296123 (PMC10849225; doi:10.1371/journal.pone.0296123)
Supplement: Attachment — Submitted filename: respose to reviewers.docx [file pone.0296123.s002.docx]

**Spatial variation and determinants of age-appropriate vaccination practice in Ethiopia using EDHS 2016 data: a spatial and multilevel analysis.**

Muluken Chanie Agimas*^1^, Meron Asmamaw^1^, Nebiyu Mekonen^1^, Fantu Mamo^1^, Daniel Alayu Shewaye^1^.

1. College of medicine and health science, institute of public health, department of Epidemiology and biostatistics, university of Gondar, Gondar, Ethiopia.

Authors address:

Muluken Chanie: [mulukensrc12@gmail.com](mailto:mulukensrc12@gmail.com)

Meron Asimamaw: [merryalem101@gmail.com](mailto:merryalem101@gmail.com)

Nebiyu Mekonen: [nebiyumek12@gmail.com](mailto:nebiyumek12@gmail.com)

Fantu mamo: [fantuma3@gmail.com](mailto:fantuma3@gmail.com)

Daniel Alayu: [danielalayu14@gmail.com](mailto:danielalayu14@gmail.com)

Corresponding Author

Muluken Chanie: [mulukensrc12@gmail.com](mailto:mulukensrc12@gmail.com)

**To: PLOS ONE <em@editorialmanager.com>**

Manuscript Number: PONE-D-23-00874

**Cover Letter for reviewer’s comments and authors responses**

We would like to thank both the Editorial board members and reviewers for kind comments and suggestions. Accordingly, we have tried to correct and respond to those comments point by point, page by page, and line by line. Therefore, we prepared this author’s response, the revised manuscript, the separate revised figures and tables, and the tracked changes of the manuscript.

Sincerely, with best regards!

Muluken Chanie Agimas

ID: [https://orcid.org/0000-0003-0808-124X](https://orcid.org/0000-0003-0808-124X?lang=en)

| **Comments from review and editorial board** | **Authors response** |
| --- | --- |
| 1. Some of your references cited in the introduction part has no relation to your document.    a. Citation #1 talks about “The impact of the vaccination program for hemorrhagic fever with renal syndrome in Hu County, China”, remove this citation and replace it with appropriate citation.    b. Citation #2 briefs about influenza vaccine not about period of vaccine administration. Revise and replace with the appropriate citations.    c. Citation #3 is not the finding of the research you cited. Rather, you used from cross reference. So, replace it with appropriate source, | Dear reviewer it is a very interesting comment and we accepted, so that we removed those references which are inappropriately cited and substituted with the appropriate references so we revised our manuscript accordingly. |
| 2. Page 3, line 58-59; revise the sentence. Next to citation 6, please add the program of vaccination based on WHO recommendation. | Great comments: totally accepted and has been revised as follows:  the world health organization (WHO) recommends that the vaccine should take **within 1-year schedule** to maximize the protective effect of the vaccine |
| 3. Page 4, line 71; write full words on their first appearance, VPDs | Great. Corrected as follows on page 4 line-69  Like other countries, in Ethiopia, age-appropriate vaccination is the key strategic activity to reduce easily preventable childhood mortality from measles, pneumonia, diarrheal diseases, and other **vaccine preventable diseases** (17). |
| 4. Page 4, line 76; the citation used for Toke kutaye reference number 18 is not correct. The reference listed on number 18 is Xiao X, Wu Z-C, Chou K-C , 2011. | Thank you dear reviewer you observe wisely and carefully. We saw the reference and really needs amendment. So the comment is corrected by the following reference  19. *Dirirsa K, Makuria M, Mulu E, Deriba BS. Assessment of vaccination timeliness and associated factors among children in Toke Kutaye district, central Ethiopia: A Mixed study. Plos one. 2022;17(1):e0262320.* |
| **Method** |  |
| 5. Page 4, line 84-85; the population of Ethiopia is 114,963,588, include your reference | Well. as long as we use another’s report we have to acknowledge the source so we admit the comment and revision has made on references and the population size of Ethiopia (latest data) |
| 6. Page 4, line 89-90; what is the need of this statement “the Ethiopian demographic health survey of 2016 revealed that among ten children aged 12-23, four children (39%) received all the basic vaccines (19).:”? | Dear reviewer it is a very interesting comment and we accepted, so that we removed those phrasing which are irrelevant to the study |
| 7. Page 6, line 119; table 1, there is no table clearly showing table 1 since table captions are 1 for all. | Great comment and correction has made by the following ways:  Intra-class coefficient (ICC) was used for the measurement of variation across regions with value of 18.7%. Analysis was undergone in four models; null model (model of without independent variables), model-I (individual level predictors), model-II (group level predictors), model-III (both model-I and model-II). For all models ICC, a proportional change in variance (PCV), log-likelihood test, and Akaike information criterion (AIC) model were calculated **(Table-1).** |
| **Results** |  |
| 8. Page 7, line 144, line 149, and page 9 line 200; you cited table 2, 3, and 4, but not appropriately used the captions. | Great view and revision also made on the table captions. |
| 9. Page 8, line 162; figure 4 is not available in lists of tables or has no caption. | Great view and revision also made on the figure-4 (figure-3 in the revised figure) caption. And the list will be incorporated when submitted |
| 10. Pages 7- 8, line 158-162; why you included about anemia? Remove it. | It is an editorial problem and the revision has made and replaced with age appropriate vaccination practice. |
|  |  |
| 11. At the end of your discussion add strength and limitation of your study. | Dear reviewers, if the journal recommends the limitation and the strength of the study as a part of the discussion, the correction has made accordingly |
| **Conclusion** |  |
| 12. Actually, you have no variable that shows lack of transportation fees. So, you could not give the following recommendation since it is not a part of your findings. So remove it. “Provide outreach vaccination for those are difficulty accessing the vaccine because of lack of transport fees could bring a significant contribution to improving age appropriate vaccination practice by health care workers.” | Okay It is obvious that the recommendations should be based on the findings. So revision has made accordingly |
| 13. Page 12, line 264-266; move the strength and limitation to discussion part. | Dear reviewers, as we have mention earlier in comment 12:  If the journal recommends the limitation and the strength of the study as a part of the discussion, we have done it. |
| **References** |  |
| 14. References 1-3 are not related with your citation, so change and update them. | Great. The necessary revision has made because the reference was unrelated and incorrectly cited. |
| 15. Reference number 19 is not fully written. Revise it. | Dear reviewer as we have stated earlier it is a very interesting comment and we accepted it. So that we removed those references which are inappropriately cited and substituted with the appropriate references so we revised our manuscript accordingly. |
| **Tables** |  |
| 16. There are 4 tables listed in the manuscript. But the caption of each table is not correct. All tables are labeled as “table 1”, revise and correct them. | Okay accepted. The caption and the labels of each table has been corrected After the correction, the revised tables and figures will be attached accordingly |
| 17. Socio-demographic table, under variable region, change Somalia to Somali. | Thank you for your detailed comments, all the comments that you have raised are very important and we just accept all the comments and we have taken a lesson from the comments. Similarly The necessary amendment has made on comment_17. Which is Somalia is changed to Somali in the revised manuscript. |
| **Figures** |  |
| 18. Out of the 7 figures listed in in this manuscript, one figure has no caption. Revise it. | Definitely. Figure-4 (figure-3 in the revised figure) has no caption. So amendment/revision has made. In the revision the most important comments of the captions are incorporated. |
| **Other comments from the reviewer** |  |
| The manuscript needs to take an editorial revision to tackle minor grammar issues. | Dear reviewer the grammar error has been reviewed. |
| To clearly indicate and just the existence of the spatial variation, it is also highly important if some spatial tests are taken into account. With these comments, I recommend that the article be published. | Dear reviewer, you are right. Definitely, if spatial tests were added it was better. But in this study, our objective was to show the geographical distribution/variation of timely vaccination practice. Therefore we have reported the finding based on the stated objectives. But to support our objective and based on your contractive and valuable comment SaT scan analysis has been added accordingly. |
| I don’t see the effects of age when dealing the spatial issues. What is the relevance of considering age as one of the explanatory variables in dealing with spatial analysis? | Dear reviewer in our research an attempt has been made to show the distribution of timely vaccination/age-appropriate vaccination across the region of Ethiopia. So age in this case shows whether the vaccine was taken at the appropriate age or not (Yes, No). For example, according to WHO, BCG, the polio-0 vaccine should take at birth, polio-1, penta-1 pcv-1 should be taken at one month or after 4 weeks and so on. Therefore in this study we show the spatial distribution of this timely/age appropriate vaccination. Not mean that the spatial variation of the child age. So age was not the explanatory variable in the study. |
| I also suggest the authors to highlight and explain the issue of spatial dependency in the methodology subsection. | Great comment. We miss it and now we revise it accordingly. |
| **Comments from editors** |  |
| In your Data Availability statement, you have not specified where the minimal data set underlying the results described in your manuscript can be found. PLOS defines a study's minimal data set as the underlying data used to reach the conclusions drawn in the manuscript and any additional data required to replicate the reported study findings in their been entirety. | Data is available online and you can access it from www.measuredhs.com |
| We note that you have stated that you will provide repository information for your data at acceptance. Should your manuscript be accepted for publication, we will hold it until you provide the relevant accession numbers or DOIs necessary to access your data. If you wish to make changes to your Data Availability statement, please describe these changes in your cover letter and we will update your Data Availability statement to reflect the information you provide. | My ID= <https://orcid.org/0000-0003-0808-124X> |
| We note that Figures 1, 4, 5, 6 and 7 in your submission contain map/satellite images which may be copyrighted. All PLOS content is published under the Creative Commons Attribution License (CC BY 4.0), which means that the manuscript, images, and Supporting Information files will be freely available online, and any third party is permitted to access, download, copy, distribute, and use these materials in any way, even commercially, with proper attribution. For these reasons, we cannot publish previously copyrighted maps or satellite images created using proprietary data, such as Google software (Google Maps, Street View, and Earth). For more information, see our copyright guidelines | Dear editor deadly sure the image never copy from other sources rather the image was from ARC GIS output. But the shape file was from CSA 2013. Therefore the source of the shape file included in the revised shape/figure. The legends of the figures also provides as an evidence for not copy from the other source.  But figure one was obtained from other sources and we cannot obtain permission from the original copyright holder to publish these figures under the CC BY 4.0 license. So we remove figure -1 |
| Please review your reference list to ensure that it is complete and correct. If you have cited papers that have been retracted, please include the rationale for doing so in the manuscript text, or remove these references and replace them with relevant current references. Any changes to the reference list should be mentioned in the rebuttal letter that accompanies your revised manuscript. If you need to cite a retracted article, indicate the article’s retracted status in the References list and also include a citation and full reference for the retraction notice. | Dear editor this comment is not only your comment but also the reviewers’. Therefore we accept the comment and an amendment has been made accordingly. |
